# Coastal Bacterial Community Response to Glacier Melting in the Western Antarctic Peninsula

**DOI:** 10.3390/microorganisms9010088

**Published:** 2021-01-01

**Authors:** María Estrella Alcamán-Arias, Sebastián Fuentes-Alburquenque, Pablo Vergara-Barros, Jerónimo Cifuentes-Anticevic, Josefa Verdugo, Martin Polz, Laura Farías, Carlos Pedrós-Alió, Beatriz Díez

**Affiliations:** 1 Department of Oceanography, Universidad de Concepcion, Concepcion 4030000, Chile; mealcaman@uc.cl (M.E.A.-A.); laura.farias@udec.cl (L.F.); 2 Center for Climate and Resilience Research (CR)2, Santiago 8320000, Chile; 3 Escuela de Medicina, Universidad Espíritu Santo, Guayaquil 0901952, Ecuador; 4 Centro de Investigación en Recursos Naturales y Sustentabilidad, Universidad Bernardo O’Higgins, Santiago 8370993, Chile; sebastian.fuentes@ubo.cl; 5 Facultad de Ingeniería, Ciencia y Tecnología, Universidad Bernardo O’Higgins, Santiago 8370993, Chile; 6 Department of Molecular Genetics and Microbiology, Pontificia Universidad Católica de Chile, Santiago 8331150, Chile; pvergar1@uc.cl (P.V.-B.); jeronimo.cifuentes@ug.uchile.cl (J.C.-A.); 7 Alfred-Wegener-Institute, Helmholtz-Centre for Polar and Marine Research, 27570 Bremerhaven, Germany; maria.josefa.verdugo@awi.de; 8 Department of Civil and Environmental Engineering, Massachusetts Institute of Technology, Cambridge, MA 02139, USA; martin.f.polz@univie.ac.at; 9 Departamento de Biología de Sistemas, Centro Nacional de Biotecnología (CSIC), Darwin 3, 28049 Madrid, Spain; cpedros@cnb.csic.es

**Keywords:** glacial melting, bacterial microbial community, coastal Antarctic zone

## Abstract

Current warming in the Western Antarctic Peninsula (WAP) has multiple effects on the marine ecosystem, modifying the trophic web and the nutrient regime. In this study, the effect of decreased surface salinity on the marine microbial community as a consequence of freshening from nearby glaciers was investigated in Chile Bay, Greenwich Island, WAP. In the summer of 2016, samples were collected from glacier ice and transects along the bay for 16S rRNA gene sequencing, while in situ dilution experiments were conducted and analyzed using 16S rRNA gene sequencing and metatranscriptomic analysis. The results reveal that certain common seawater genera, such as *Polaribacter*, *Pseudoalteromonas* and HTCC2207, responded positively to decreased salinity in both the bay transect and experiments. The relative abundance of these bacteria slightly decreased, but their functional activity was maintained and increased the over time in the dilution experiments. However, while ice bacteria, such as *Flavobacterium* and *Polaromonas*, tolerated the increased salinity after mixing with seawater, their gene expression decreased considerably. We suggest that these bacterial taxa could be defined as sentinels of freshening events in the Antarctic coastal system. Furthermore, these results suggest that a significant portion of the microbial community is resilient and can adapt to disturbances, such as freshening due to the warming effect of climate change in Antarctica.

## 1. Introduction

As a consequence of climate change, polar regions are the most radically changing places on the planet [1,2]. Over the past 50 years, the Western Antarctic Peninsula (WAP) has warmed four times faster than the average rate of overall warming on Earth [3,4]. This accelerates the collapse of ice shelves, the retreat of glaciers and the exposure of new terrestrial habitats [2,4]. Climate models suggest a substantial retreat of the West Antarctic Ice Sheet [5]. A further increase of 0.5 °C beyond the present-day average global surface seawater temperature will lead to multiple impacts on a variety of organisms, from phytoplankton to marine mammals [6,7,8,9]. Significant surface freshening has been observed in the WAP close to coastal glacial meltwater sources [10,11], increasing the discharges of nutrients, metals, particles and freshwater microorganisms from glacial and sea ice meltwater to the coastal marine ecosystem [12,13,14,15]. Consequently, large phytoplankton blooms, which support heterotrophic microbial production, are expected to increase during the short austral summer [13,16,17]. The freshening and warming of the coastal surface seawaters will reduce surface seawater salinity and density, resulting in water column stratification and increased irradiance levels in shallow surface waters [12,18]. Hence, geophysical processes will force extensive changes to marine organisms by abruptly and permanently changing the physical environment, which will have significant consequences for the structure and function of existing microbial communities and biogeochemical cycles [19].

Surface bacterioplankton communities show considerable variability between different areas of Antarctica. However, Bacteroidetes, Gammaproteobacteria, Alphaproteobacteria and Actinobacteria are generally the dominant bacterial taxa in the deep subsurface shelf waters throughout the year [20,21,22,23,24,25]. During the austral summer in the WAP, bacterioplankton appear to be dominated by chemoheterotrophs, such as the *Roseobacter* clade and Gammaproteobacteria (i.e., *Shewanella*, *Vibrio* and *Oceanospirillum*), photoheterotrophs (i.e., SAR11, SAR92 and *Polaribacter*), and aerobic anoxygenic phototrophs (Rhodobacterales and Gammaproteobacteria), while autotrophic carbon assimilation is mainly driven by small oxygenic photoautotrophic eukaryotes [21,22,24,26,27]. Changes in the bacterial diversity have been observed after increases in the chlorophyll *a* (Chl *a*) concentration during summer blooms, where Gammaproteobacteria (i.e., *Alteromonas*, *Pseudoalteromonas*, *Marinobacterium* and Oceanospirillales), Alphaproteobacteria (i.e., *Roseobacter*, *Candidatus*, *Pelagibacter* and *Planktomarina*) and Bacteroidetes (i.e., *Polaribacter*) have the largest relative abundances and activity [24,25]. Conversely, SAR11, *Sulfitobacter* and some Rhodobacterales (i.e., *Octadecabacter*) clades of Alphaproteobacteria, together with some Gammaproteobacteria (mostly *Psychromonas* and *Pseudoalteromonas*), seem to be prevalent during both the summer and winter seasons [21,28,29].

Climate change will likely result in more ice melting and this, in turn, will increase water column stratification, which has been shown to have an impact on micro-eukaryotic and bacterial community composition and primary production [27,30,31]. A decrease (~12%) in phytoplankton bloom occurrence has been estimated latitudinally along the northern WAP due to increased glacial melt, retracted sea ice coverage, a greater number of cloudy days, larger upper mixed layer depth and stronger wind intensity [32,33]. Changes that depend on the climate and local physical conditions of the coastal seawaters can affect the metabolic capabilities of the indigenous microorganisms, which could trigger shifts in the community composition and dynamics. However, the way in which different geophysical and biogeochemical scenarios in marine polar environments develop and are affected by climate change remains poorly understood due to a lack of baseline knowledge regarding the microbial diversity and interactions under the current conditions.

In the present study, the microbial community composition along a surface salinity gradient in the coastal waters and surrounding glacier ice at Chile Bay (Greenwich Island) in the WAP were analyzed using 16S rRNA gene sequencing. Additionally, in situ dilution experiments were carried out, with the most active bacteria and their metabolic capacities evaluated using metatranscriptomics. These approaches generated the first example of comprehensive data that explores the effect of (i) ice–freshwater discharge and (ii) ice–microorganism transport into coastal systems due to glacial melt and consequent freshening. This information contributes to the understanding of how ecological shifts in response to specific factors such as salinity will affect specific members of the microbial community. Ultimately, this will provide information as to how the accelerating reduction of ice cover and consequent freshening of coastal waters will potentially influence the microbial communities in coastal Antarctic waters.

## 2. Materials and Methods

### 2.1. Sample Collection along the Salinity Transect

**Figure 1:**
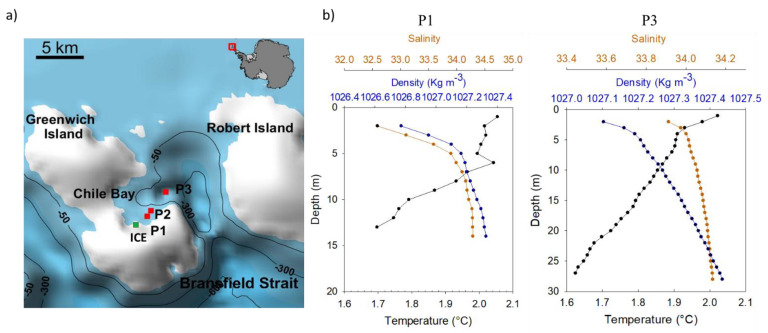
(**a**) Location of Chile Bay on Greenwich Island and stations P1 to P3 [53]. The green square indicates the glacier ice sampling location at Fuerza Aérea Glacier. (**b**) Profiles of physical variables at P1 and P3 stations showing vertical changes in temperature (°C), salinity and density (Kg m^−3^).

Chile Bay is located on the north side of Greenwich Island in the South Shetland Islands, WAP (62°27′6′′ S, 59°40′6′′ W). This Antarctic expedition was undertaken from 18th February to 4th March 2016. Seawater samples were collected from the surface layer (2 m depth) at three different stations across an in situ salinity gradient in the bay (Figure 1). Station P1 (salinity 32.5, as a result of glacier melting into the surface seawater; halocline at 5–7 m depth) was close to the nearby glacier (approximately 0.5 km of the station lays the Fuerza Aerea glacier), station P2 (salinity 34.0) was an intermediate site and station P3 (salinity 34.2) was at the mouth of the bay. Profiles of temperature (°C), salinity and density (kg m^−3^) at distant stations P1 and P3 were obtained using a SeaBird19 CTD. Seawater (20 L) was collected at the three stations using a manual pump on board a Zodiac boat. The water samples were directly deposited into previously cleaned bottles (10% HCl) and then transported to the laboratory at the Chilean station Captain Arturo Prat (Instituto Antártico Chileno), Chile Bay. Triplicate seawater subsamples for nutrient analysis were filtered with 0.7 µm GF/F glass fiber filters, placed in 15 mL polypropylene tubes and stored at −20 °C until laboratory analysis. The final concentration of nitrite (µM) (detection limit 0.05 μM), nitrate (µM) (detection limit 0.05 μM), phosphate (µM) (detection limit 0.02 μM) and silicate (µM) (detection limit 0.65 μM) were obtained by standard colorimetric assays [34], and detected using an autoAnalyzerII/3TM, TrAAcs (Seal, Mequon, WI, USA) [24] which consists of four channels with specific modules for each nutrient. The chlorophyll *a* concentration was determined in triplicate by collecting 1 L of seawater on a 0.7 µm GF/F glass fiber filter. Next, the contents of the frozen filters were extracted with acetone (90%) and then the extract was measured fluorometrically [35]. Small pieces of glacier ice (ICE) were collected from the Fuerza Aérea glacier (Figure 1a), ensuring no contamination with soil or seawater. The ICE was then transported to the laboratory in a sterilized box and maintained at room temperature (~4 °C) for 24 h until it melted. The nutrients, Chl *a* and nucleic acids were collected from the melted ice water using the same protocol described for seawater.

### 2.2. Dilution Experiments and Experimental Setup

Dilution experiments were conducted to simulate decreased salinity due to glacier ice melt. Prefiltered (150 µm polyester mesh to discharge large microorganisms) surface seawater (SW) from station P3 was mixed with melted ICE in clear polycarbonate bottles (20 L) to reduce the salinity from 34.2 to 24.0, which represents the typical salinity found in Subantarctic continental areas surrounded by glaciers and rivers, such as those in Chilean Patagonia. Two different microcosm conditions were performed: SW+ICE (salinity 24.0, with marine and ice microorganisms) and SW+ICE-F (salinity 24.0, with only marine microorganisms) (Table S2). Additionally, SW (P3) and melt ICE microcosms were conducted separately as controls. All four microcosm experiments were maintained at the in situ temperature (0–2 °C) for 1 day (t1) and a different set for 7 days (t7, final time of the experiment). Triplicate subsamples were collected from each bottle to measure nutrients (15 mL; nitrite, nitrate, phosphate and silicate) and chlorophyll*-a* (1 L) at t1 (24 h) and t7 (7 d). To analyze the microbial community diversity changes, 3 L subsamples were taken at t1 and t7 (final time) from all bottles to extract DNA for subsequent 16S rRNA gene sequencing. An additional 3 L were collected from each bottle at t7 for RNA extraction and metatranscriptomic analysis to determine the most active bacterial components and metabolic changes potentially related to the salinity shift perturbation.

### 2.3. Microbial Biomass Concentration and Nucleic Acid Extractions

For in situ microbial biomass concentration and subsequent DNA/RNA extractions, seawater samples were collected along the salinity gradient at stations P1, P2 and P3 in Chile Bay, as well as for the melted glacier ice collected near the bay. For each seawater sample, 3 L of prefiltered seawater and melted glacier ice (150 µm polyesters mesh) was passed through a 0.22 µm pore size PES Sterivex filter (Millipore, Darmstadt, Germany) using a Cole Palmer peristaltic pump system (600 rpm). To preserve the integrity of the RNA, 1 mL of RNAlater RNA Stabilization Solution (Ambion^TM^) was added to each filter before storage. All filters (both DNA and RNA) were immediately frozen in liquid nitrogen until further analysis in the laboratory. The same procedure was performed for the melted glacier ice samples and the dilution microcosms experiments.

DNA extraction was based on the method described by [24]. Briefly, filters were resuspended in lysis buffer and sterile glass beads (1 mm; Sigma Aldrich, Darmstadt, Germany) and then incubated at 37 °C for 1 h. DNA was extracted with phenol-chloroform–isoamyl alcohol (25:24:1), and then the residual phenol was eliminated with chloroform–isoamyl alcohol (24:1). The DNA was cleaned using two successive cold isopropanol precipitations with an ethanol (70%) wash.

For RNA extractions, each RNA filter was cleaned of RNAlater by successive washes with sterilized ddH_2_O. Next, 1 mL of TRIzol (Invitrogen, Waltham, MA, USA) was added; then, the mixture was subjected twice to 20 s of bead-beating. An RNA Clean & Concentrator kit (Zymo Research, Orange, CA, USA) was then used according to the instructions. Finally, the quality and quantity of the total extracted nucleic acids were checked with a Qubit fluorometer (Invitrogen) and a dsDNA-BR Assay kit for DNA or an RNA-BR Assay kit for RNA. The integrity was checked via 0.8% agarose gel (DNAse/RNAse-free) electrophoresis.

### 2.4. 16S rRNA Gene Amplification, Sequencing and Taxonomic Annotation

16S rRNA gene i-Tag sequencing was performed on DNA samples from stations P1 (4), P2 (1) and P3 (6) during 18th February to 4th March 2016, and on samples t1 and t7 and the controls (SW+ICE; SW+ICE-F; SW; ICE) from the dilution microcosms experiments. Amplification and sequencing of the 16S rRNA gene was performed according to the Earth Microbiome Project protocols (www.earthmicrobiome.org/protocols-and-standards). The V4-V5 hypervariable region of the 16S rRNA gene was amplified by PCR using the bacterial–archaeal primer pairs 515F (5′GTGYCAGCMGCCGCGGTAA 3′) and 926R (5′CCGYCAATTYMTTTRAGTTT3′) [36]. The amplification products were multiplexed and sequenced on the Illumina MiSeq platform (250 bp × 2) at Argonne National Laboratory (Lemont, Chicago, IL, USA). The sequences have been submitted to the NCBI BioProject database under project identification number PRJNA663269 and sample accession numbers SAMN16124624 to SAMN16124642.

Processing of the 16S rRNA gene sequences was performed with the QIIME2 package v.2018.11 [37] and the software within. Sequences were quality-checked, assembled and chimera-filtered using the DADA2 algorithm [38], which defines the operational taxonomic units at 100% identity, i.e., amplicon sequence variants (ASVs). Taxonomy of each ASV was assigned using the SILVA 132 database [39]. Reads classified as chloroplasts (14.8% in total) were filtered into a separate data set, which was then classified using the plastidial 16S rRNA database PhytoRef (http://phytoref.sb-roscoff.fr, downloaded on 8 October 2020). Both assignations were done with the classify-consensus-vsearch plugin implemented in Qiime2. Reads classified as mitochondria (1.8%) were discarded. The final Bacteria/Archaea table was composed of 339,220 reads distributed into 893 ASVs across the 19 samples (*n* = 4 P1, *n* = 1 P2, *n* = 6 P3, *n* = 2 glacier ice and *n* = 6 microcosms). The plastid table was composed of 60,062 reads distributed into 76 ASVs across the same 19 samples.

### 2.5. Metatranscriptomic Taxonomy and Metabolic Annotation Analyses

Between 700 ng and 1 µg of clean total RNA extracted from the control (SW and ICE), SW+ICE and SW+ICE-F microcosms experiments at the final time (t7) were sequenced for metatranscriptomic analysis via Illumina Hi-Seq technology (DNA Sequencing & Genotyping Center, Delaware, Newark, DE, USA). Briefly, the total RNA was processed using standard procedures and depleted of rRNA using RiboZero. The RNA-Seq libraries were prepped using NEXTflex Rapid Directional RNA-Seq Kit from Bioo Scientific. The quality of the metatranscriptomic raw reads was assessed with FastQC (Andrews, 2010). The sequences were quality filtered using Cutadapt [40]. The first nine leftmost bases were removed and 3′ trimming of bases with quality below 30 was performed. The remaining rRNA sequences were removed using SortMeRNA software [41] with SILVA (version 132) as a reference database [39].

#### 2.5.1. Marine Microbial Protein Database Construction

The principal genes involved in energetic metabolism associated with carbon fixation and nitrogen assimilation were searched for in the dilution microcosm experiments.

Prodigal [42] (−p meta −n) was used to predict bacterial and archaeal protein sequences from previously reported marine metagenome-assembled genomes (MAGs) recovered from the metagenomic dataset of the TARA Oceans project [43]. *Polaromonas*, *Polaribacter* and *Flavobacterium* proteins where specifically obtained from several genomes recovered from the Genome Taxonomy Database (GTDB; release 89), due to their low representation in the TARA Oceans MAGs [43]. Taxonomy of the bacterial and archaeal MAGs was inferred using GTDBtk (release 89) [44].

Eukaryotic proteins and their taxonomic identity were obtained from the MMETSP project [45]. Functional protein identities were assigned to the best candidate ortholog using eggNOG-mapper software [46,47] (−m diamond), which obtained the potential KEGG ortholog (KO) identity for each protein. Additional functional information was analyzed with HMMER [48] (hmmscan) using the Pfam-A database [49]. Taxonomic and functional identity was parsed using the R program.

#### 2.5.2. Metatranscriptomic Transcriptional Level Analysis

To obtain the microbial gene transcriptional levels, non-rRNA metatranscriptomic reads obtained from microcosms (SW and ICE controls, and SW+ICE and SW+ICE-F dilution experiments) at the t7 final time were recruited to our custom protein database using DIAMOND [50] with an E-value of 1 × 10^−8^, and then assigned to the best hit. The transcript levels from the four samples were normalized by quantiles using the R package preProcesscore [51]. As eggNOG orthologs can have more than one KO identity, the transcript level per microbial gene was penalized by dividing it by the number of KOs assigned to each microbial protein to prevent read overcounting due to multiple KO assignments, similar to [52]. The final transcriptional levels from each microcosm sample were calculated by adding up the penalized counts from each KO per taxa, using the R package Tidyverse (https://www.tidyverse.org/). Finally, the KOs were organized into pathways using information from the KEGG website. The KO and Pfam accession IDs for the carbon and nitrogen cycles were used to analyze these specific pathways in this study and are listed in Annex 1. All scripts used in this work are available upon request. This data has been deposited in the NCBI database under accession number PRJNA663269 and sample accession numbers SAMN16124624 to SAMN16124642.

## 3. Results

### 3.1. Environmental Conditions

During the late summer (February) of 2016, three different stations (P1, P2 and P3) along an in situ surface salinity gradient (32.5 to 34.2) were sampled in Chile Bay, WAP (Figure 1a). Additionally, glacier ice from a glacier close to Chile Bay was sampled to represent the freshwater input responsible for the salinity decline in these coastal seawaters.

Surface seawater temperature remained around 2.0 °C along the transect and decreased with depth down to 1.6 °C (Figure 1b). A surface mixed layer was present, with the halocline at 2–3 m deep at P3 and 5 m deep at P1 (Figure 1b).

Nutrient concentrations in the glacier ice samples were exceptionally low in comparison to those of the seawater sites (Table S1). Nitrite and phosphate were in the order of 0.138 ± 0.004 µM and 0.158 ± 0.03 µM, respectively, while nitrate and silicates were around 0.776 ± 0.05 µM and 0.225 ± 0.03 µM, respectively. In contrast, seawater samples were one order of magnitude higher in nutrient concentrations, with a slight increase offshore at P3 (Table S1). Chl *a* concentrations were also low in the glacier ice at around 0.291 ± 0.02 mg m^−3^, while they were one order of magnitude higher in the seawater, with an average value of 3.65 mg m^−3^ (Supplementary data; Table S1).

In the dilution experiments (Table S2), all nutrients decreased around 18% to 31% relative to the SW control after the first 24 h (t1) due to dilution and biological activity (Table S2). Likewise, Chl *a* experienced a slight decrease compared to the SW t1. After 7 days (t7) of incubation, the SW control showed a high decrease of nitrite (33%), nitrate (67%), phosphate (55%) and silicate (44%). However, in the SW+ICE microcosms, the most notably decreased nutrients were nitrate (32%) and phosphate (34%), and in the SW+ICE-F the same nutrients decreased 23% and 30%, respectively (Table S2). Contrary to the nutrients, Chl *a* at t7 increased 14-fold in SW (23.5 mg m^−3^) and SW+ICE-F (16.1 mg m^−3^), and 18-fold in SW+ICE (17.1 mg m^−3^), denoting high biological activity inside the microcosms after 7 days of incubation (Table S2).

### 3.2. Changes in Bacterial Composition

**Figure 2:**
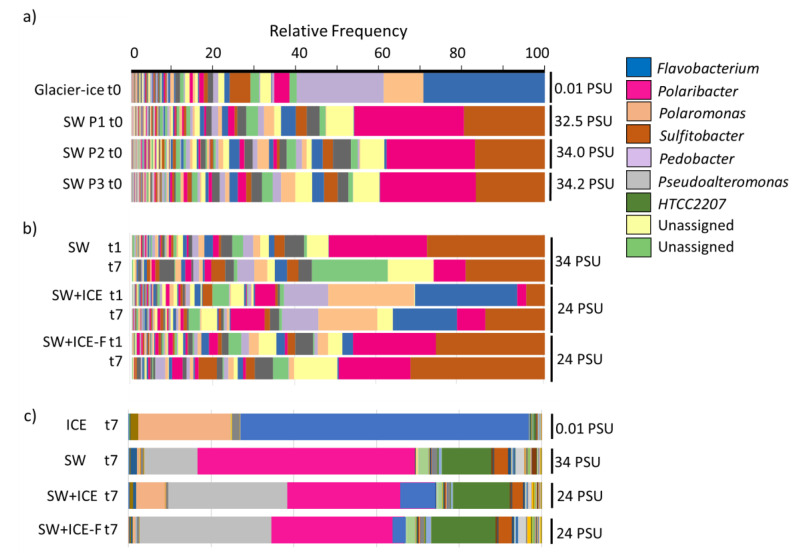
Bacterial composition in Chile Bay (**a**) and the dilution experiments (**b**,**c**). (**a**) 16S rRNA i-Tag sequencing of glacier ice and stations P1, P2 and P3. (**b**) 16S rRNA i-Tag sequencing of t1 and t7 from the dilution experiments, and the SW control. (**c**) Metatranscriptomic reads at the final time (t7) for the glacier ice (ICE) and surface seawater (SW) controls, and the SW+ICE and SW+ICE-F dilution experiments. Time 0 (t0) of the experiments is represented by the in situ composition (**a**). The legend indicates the most abundant genera representatives according to the relative percentage abundance.

The bacterial composition is summarized in Figure 2. The glacier ice community was dominated by Bacteroidetes (61.2% of the reads) and Proteobacteria (30.8%). Most Bacteroidetes belonged to Flavobacteriales (31.3%), with a high dominance of the genus *Flavobacterium* (29.3%), and Sphingobacteriales (22.3%), particularly the genus *Pedobacter* (20.9%). Burkholderiaceae (22.1%), mostly represented by *Polaromonas* (9.6%), was the most abundant proteobacterial group. The seawater microbial community was also dominated by Bacteroidetes (P1: 51.2%; P2: 47.3%; P3: 50.4%) and Proteobacteria (P1: 43.1%; P2: 46.2%; P3: 44.9%). However, taxonomic affiliations showed *Polaribacter* (Flavobacteriales; Pl: 26.4%, P2: 21.3%, P3: 23.0%) as the most abundant Bacteroidetes. Within Proteobacteria, Alphaproteobacteria (P1: 30.1%; P2: 27.8%; P3: 30.9%), especially *Sulfitobacter*, was the dominant group rather than Gammaproteobacteria (P1: 12.7%; P2: 17.3%; P3: 13.9%). The relative abundance of *Sulfitobacter* (Alphaproteobacteria, Rhodobacteraceae) decreased slightly from P1 (19.5%) to P3 (16.6%), showing a similar pattern as *Polaribacter* (Figure 2a).

We also analyzed the taxonomic composition of the 16S rRNA gene reads belonging to chloroplasts of eukaryotes (Figure S1). The phytoplankton community was clearly different between the glacier ice and seawater. Prasiolales and Vaucheriales dominated the glacier ice, while Bacillariophyceae were predominant in the seawater (Figure S1). Archaea were not detected in the 16S rRNA gene sequencing, despite the fact that the primers used largely cover this group.

For the dilution experiments (Figure 2b), 16S rRNA gene sequencing was used to analyze the composition at the initial (t1) and final (t7) times, while metatranscriptomic analysis (Figure 2c) was performed only at t7. The main bacterial representatives in the natural SW experienced changes in their relative abundances through time (Figure 2b). In particular, *Polaribacter* and *Sulfitobacter* decreased, while unassigned genus reads (yellow and light green in the figure belonged to the Flavobacteriales order) increased with time. The glacier ice community dominated the SW+ICE experiment at t1 (Figure 2b). *Flavobacterium* represented 24.6% at t1 and declined to 15.5% at t7, *Polaromonas* represented 20.7% (t1) and decreased to 14.2% at the final time and *Pedobacter* showed minor changes from t1 to t7 (from 10.7% to 8.8%, respectively) (Figure 2b). A low abundance of seawater members, such as *Polaribacter* (2.2%) and *Sulfitobacter* (4.5%), was detected at t1, but these community members increased by t7, reaching 6.8% and 14.3%, respectively. As expected for the SW+ICE-F experiment (Figure 2b), the seawater bacterial community dominated overtime, with the typical seawater members *Polaribacter* and *Sulfitobacter* accounting for 19.4% and 26.2%, respectively, at t1, and 17.3% and 32.4%, respectively, at t7, similar to the SW control. However, while *Polaribacter* decreased by 33% over time in the SW control, it only decreased 10.8% by t1 (19.4%) and t7 (17.3%) in the SW+ICE-F experiment. In turn, *Sulfitobacter* increased by 24% at t7 (32.4%) with respect to the initial time (26.2% (t1)) (Figure 2b).

Metatranscriptomic analysis (Figure 2c) revealed that in the ICE, *Flavobacterium* and *Polaromonas* were the most active members, while *Pedobacter* did not exhibit much activity. In the three seawater experiments, *Polaribacter* was the most active genus at the final time, showing 14% lower activity in the dilution experiments. Conversely, *Pseudoalteromonas* showed higher expression levels in the dilution experiments. Two other bacteria showed opposite patterns: HTCC2207 exhibited relatively high expression in contrast to its low abundance in all SW-containing incubations, while *Sulfitobacter* had low activity (Figure 2c) despite its abundance in the seawater (Figure 2a,b).

### 3.3. Active Response of Microbial Assimilatory Carbon and Nitrogen Pathways to Freshening

As expected, expression patterns of the 1000 most active genes from Bacteria, Eukarya and Archaea revealed different profiles for the glacier ice community relative to that of the SW and dilution experiments (Figure S2). Notably, the Archaea domain was the least represented, and thus was excluded from the functional analysis.

A previous study in Chile Bay [24] revealed high rates of carbon fixation and inorganic nitrogen assimilation. Therefore, we paid special attention to the main active genes involved in these two assimilation pathways. All genes considered in this analysis are listed in Annex 1.

**Figure 3:**
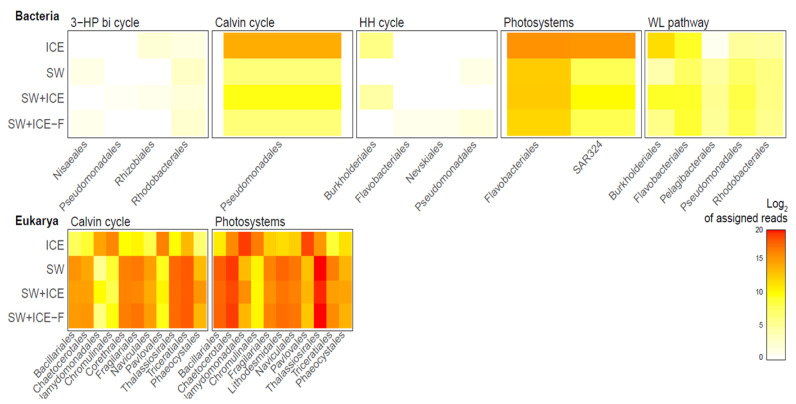
Active microorganisms (based on transcript numbers) in the carbon fixation pathways, in response to dilution (SW+ICE and SW+ICE-F microcosms), compared to the ICE and SW controls. Key genes related to the 3-hydroxypropionte bicycle (3-HP bicycle), hydroxypropionate-hydroxybutyrate cycle (HH cycle), reductive acetyl-CoA (WL pathway), Calvin cycle and photosystems were evaluated jointly with the most active Bacteria and Eukarya representatives. The values are expressed as Log_2_ of the assigned reads.

As could be expected, Calvin cycle and photosystem genes were mostly derived from active eukaryotes (Figure 3). In the ice, active groups such as Chlamydomonadales, Chromulinales and Pavlovales were most prevalent, while Bacillariales, Chetocerales and Thalassiosirales, among others, were more active in the dilution experiments. Contributions of bacteria to these processes were minor (<3% of the reads) compared to the eukaryotic community and due exclusively to Pseudomonadales, Flavobacteriales and SAR324, that were mostly found at the ice (Figure 3). A few bacterial groups (<0.1% of the reads) showed some activity in the Wood–Ljungdahl pathway, while both the 3-hydroxypropionate bicycle and the hydroxypropionate-hydroxybutyrate cycle were practically inactive. In brief, most inorganic carbon fixation was achieved through oxygenic photosynthesis, which was attributable to eukaryotic phytoplankton.

**Figure 4:**
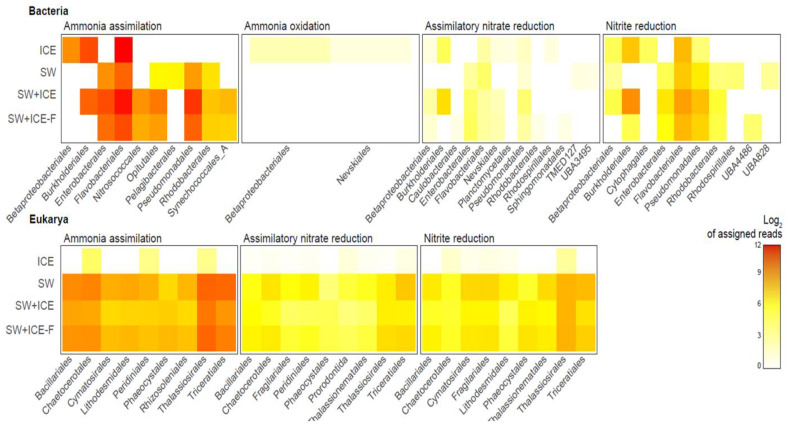
Active microorganisms (based on transcript numbers) in the nitrogen assimilation pathways for the dilution experiments (SW+ICE and SW+ICE-F microcosms) and controls (SW and ICE). Key genes related to ammonia assimilation, ammonia oxidation and assimilatory nitrate and nitrite reduction were evaluated together with the most active bacterial and eukaryotic representatives. The values are expressed as Log_2_ of the assigned reads.

Conversely, bacteria and eukaryotes showed different nitrogen assimilation strategies (Figure 4). Eukaryotes were equally active in ammonia and nitrate uptake, while bacteria preferred ammonia over nitrate. Eukaryotes were more active in the water samples than in the ice, while bacteria showed activity in both sample types. Among the latter, Betaproteobacteria, Burkholderiales and Flavobacteriales were more active in the ice, while Enterobacteriaceae, Flavobacteriaceae and Pseudomonadales were especially active in the seawater samples.

### 3.4. Active Microbial Sentinels in Response to Freshening

**Figure 5:**
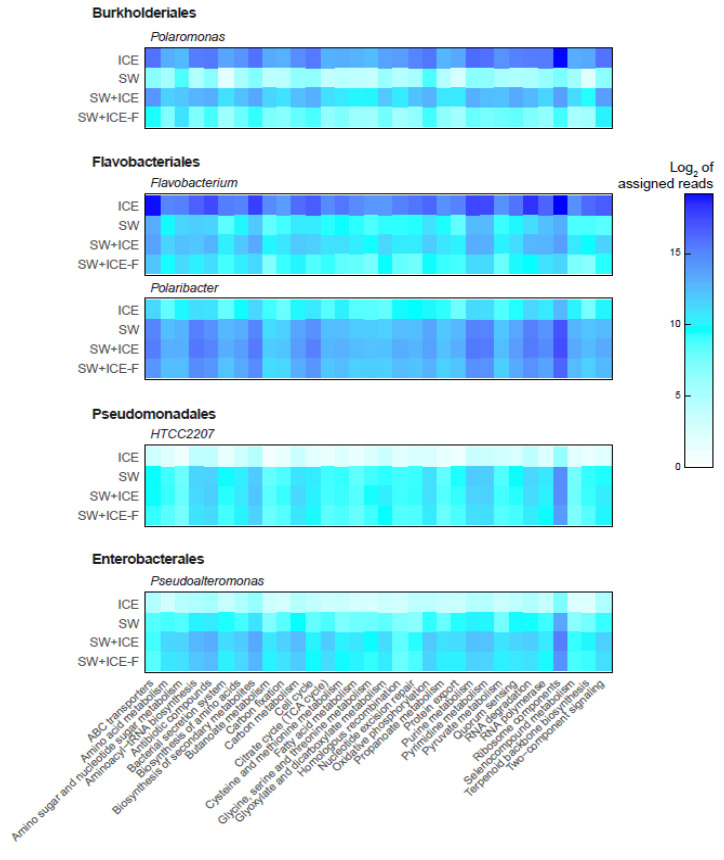
The most active metabolic functions of bacterial taxa from glacier ice and seawater. *Polaromonas* and *Flavobacterium* are glacier ice representatives, while *Polaribacter*, HTCC2207 and *Pseudoalteromonas* are marine relatives. The values are expressed as Log_2_ of the assigned reads.

The most active bacterial taxa in the glacier ice were *Flavobacterium* and *Polaromonas*, while those in SW were *Polaribacter*, *Pseudoalteromonas* and Pseudomonadales HTCC2207. We considered these five bacteria as potential sentinels of freshening in this coastal system; thus, we examined the most active metabolic functions (>30; Figure 5) that were shared by all of them.

*Polaromonas* showed a certain tolerance (with a 7-fold decrease in activity) to live when glacier ice was mixed with SW (SW+ICE). The functions that most decreased were ribosome components (40-fold), RNA polymerase (11-fold), terpenoid backbone biosynthesis and homologous recombination activities. *Flavobacterium*, another glacier ice representative, in general showed a 24-fold decrease in the activity of metabolic functions in SW+ICE relative to the ICE control; a 40-fold decrease in expression of genes for ABC transporters, ribosome components and RNA degradation; and a more than 90-fold decrease in terpenoid backbone biosynthesis activity (Figure 5).

Three seawater bacteria were found to partially tolerate the salinity dilution (Figure 5). Even though all activities decreased, no remarkable specific changes were observed in any of the most active C and N pathways analyzed. *Polaribacter* accounted for the majority of the reads from marine representatives, not changing under the dilution conditions. The trend observed for *Polaribacter* was similar to that of Pseudomonadales bacterium HTCC2207. This bacterium responded positively, doubling its activity for carbon, glyoxylate and dicarboxylate metabolism, and for two-component signaling in the SW+ICE treatment. In turn, the activity of *Pseudoalteromonas* increased on average by 6-fold for most functions in the SW+ICE dilution experiment, and 4-fold in SW+ICE-F (Figure 5). The most marked increase (7-fold) in transcripts of this bacterium was observed for carbon metabolism, citrate cycle (TCA cycle), fatty acids, antibiotic compounds and biosynthesis of secondary metabolites in the SW+ICE microcosm.

## 4. Discussion

### 4.1. Chile Bay: A Coastal WAP Scenario to Test Microbial Community Response to Freshening

The West Antarctic Peninsula (WAP) region has undergone significant changes in temperature and seasonal ice dynamics, with substantial impacts on the regional ecosystem, ocean chemistry and hydrographic properties [4,7,54]. Antarctica’s continental margins, due to the melting of glacial ice and snow, contribute freshwater to the surface seawater, breaking down the surface salinity. This low-salinity meltwater has been linked to increased phytoplankton blooms in nearshore waters [55]. In the WAP, sea ice and glacier melting are important physical determinants of spatial and temporal changes in microbial communities [12]. The stratification and transition to non-stabile surface waters impact both microeukaryotic and bacterial community compositions [30]. Recently, Hofer et al. [31] correlated surface freshwater plumes in Maxwell Bay and South Bay, WAP, with high phytoplankton growth rates, suggesting that these bays act a source of organic matter to neighboring oceanic waters.

Chile Bay in the WAP is considered a highly productive system during the austral summer, exhibiting seasonal changes in the bacterial and eukaryotic communities [24,25]. Although water column mixing is highly dynamic due to the strong winds that hit this coastal area, glacial melt into Chile Bay seems sufficient to establish slight stratification in the first five meters of the water column (Figure 1b).

In order to understand the response of marine microbial communities in Chile Bay to natural freshwater input from glacier melting, we analyzed changes in the relative abundances of marine bacteria along a transect from the glacier towards offshore marine waters of the bay during the summer of 2016. Our study demonstrates the presence of a diatom phytoplankton bloom during the 2016 summer sampling, with similar Chl *a* concentrations (up to 4 mg m^−3^) as those previously reported for the late summer of 2014 [24]. We demonstrate that in 2016, *Polaribacter* exhibited a slight reduction in their abundance further offshore where the seawater is more saline; however, *Polaribacter* was one of the most dominant members in terms of relative abundance at near-glacier sites where the salinity is lower, suggesting high adaptation to freshening. *Polaribacter* has been previously suggested to originate from melting sea ice in the Arctic and Antarctica [21,56], where, upon the melting of spring ice, this genus colonizes the marine water column and frequently becomes dominant in polar waters along with phytoplankton blooms [57]. This might explain the high *Polaribacter* abundances found in our seawater samples during the summer of 2016, while we only detected a low abundance (<5%) in the glacier ice samples. Another dominant seawater taxon was *Sulfitobacter*, a *Roseobacter*-clade member that is considered a predominant bacterioplankton in the Antarctic Peninsula [58], and a common marine and polar sulfite oxidizer [59,60,61]. Similar to *Polaribacter*, the abundance of this bacterium slightly decreased as the transect became saltier. Although the prevalence of these two genera has been described as seasonal and variable between poles [21,28,62], it is established that both can coexist in coastal marine systems (Grzymski et al., 2012), as was observed here for Chile Bay.

Moreover, the glacier ice microbial community near Chile Bay exhibited dominance of the phyla Proteobacteria and Bacteroidetes, as previously reported for other glacier samples, such as those from the Svalbard Archipelago [63]. Here, the most dominant bacterial members of the ice community were *Flavobacterium*, *Pedobacter* and *Polaromonas*, which have also been widely reported in other glacier ice ecosystems [63,64,65]. *Flavobacterium*, the most dominant genus, is widespread in nature and has been isolated from many freshwater and soil habitats, including polar systems. This bacterium plays a crucial role in the remineralization processes and exhibits strong macromolecular hydrolytic capabilities [66]. Besides these genera, other ice-associated members, such as Rhodobacterales and Sphingobacteriales, were detected in seawater closest to the Chile Bay glaciers, suggesting a contribution from glacial freshening into these coastal seawaters of Antarctica. However, these taxa were not as abundant as other marine bacteria within the costal system, suggesting low adaptation or potential competition with marine bacteria. However, more studies will be necessary to test this hypothesis.

Altogether, our results suggest that major seawater representatives show subtle adaptation to the decreased surface salinity in Chile Bay due to local glacier melting. These findings led us to connect the bacterial composition and abundances of these two environments in order to understand how the bacterial community changes when faced with natural freshening events. In this way, Chile bay is an ideal natural coastal site in Antarctic marine waters to test how climate change is affecting the WAP in relation to prior effects on terrestrial environments, such as glacier melting. Microbial sentinels, for those types of effects, are now needed to fully understand the impact that climate change is having in the region.

### 4.2. Bacterial Sentinels in Response to Manipulated Freshening

In order to confirm some of our previous mentioned results obtained from natural waters of Chile Bay, dilution experiments with Chile Bay marine waters were performed. It is a fact that the WAP has been subject to periods of warming surface temperatures and that enhanced mass loss from melting glaciers has triggered consequences for the functional characteristics of the whole ecosystem [67,68]. Here, we evaluated the effect of seawater dilution, similar to that observed in the natural seawater of the sub-Antarctic region (22–24 salinity units) [69,70], and the Svalbard Archipelago [63], mimicking the magnitude of thaw on the Peninsula if we fail to slow the present speed of global warming. Garcia-Lopez et al. [63] showed an interchange between glacier and coastal microbial populations in Norwegian fjords, identifying the presence of some indicator species as possible sentinels for bacterial transport between glaciers and the downstream seawater. Although composition and abundance changes are often investigated, microbial activities have not been addressed in this type of study, which is needed to fully understand how these sentinels have adapted to the new scenarios of climate change in the affected polar regions.

The results of our dilution experiments revealed the presence of several taxa that could be used as potential sentinels, such as *Polaribacter*, which live and subsequently thrive in terms of relative abundance, maintaining important gene expression after seven days in the dilution microcosms (Figure 2b,c). *Polaribacter* is considered the most common and significant psychrophilic genus of the Southern Ocean, associated with high nutrients and eukaryotic algal primary production [62], but it is also recognized as widely distributed in other cold and temperate marine habitats [71]. Both *Polaribacter* and *Polaromonas* have been previously proposed as sentinel microorganisms for the exchange of materials from glaciers to seawater [63]. Despite the fact that the *Polaribacter* and *Polaromonas* genera showed decreased global transcription with respect to the SW and ICE controls in the dilution experiments (Figure 2c), they behaved as good sentinels since they did not show remarkable gene expression changes in the most expressed pathways under the dilution conditions (Figure 5). However, among all possible seawater sentinels, *Polaribacter* was unique in that ABC transporter expression increased (up to 28%) in response to dilution (Figure 5). ABC transporters are ubiquitous in bacteria and function as importers of growth substrates and factors, including carbohydrates, amino acids, polypeptides, vitamins and metal-chelate complexes [72]. They are also considered as good indicators of bacterial DOM utilization patterns [73]. In this sense, the relative abundance of *Polaribacter* has been previously reported to increase in response to DOM addition in mesocosms from coastal WAP systems [74]; therefore, the increased ABC transporter expression in our experiments might be related to the bioavailability of DOM, which favors the positive performance of this genus in both seawater and diluted conditions.

*Sulfitobacter*, the other dominant genus found in Chile Bay seawater, exhibited increased abundance at the end our dilution experiments, even though the gene expression was minimal after seven days (Figure 2c). In both diluted and undiluted incubations, the behavior of *Sulfitobacter* indicates that this pattern was not related to the salinity dilution, but perhaps to a bottle effect during the experiment. Therefore, using this taxon as a potential sentinel should be reevaluated with further experiments to fully understand the effect of freshening on this genus. This behavior could also be explained by niche competition with other DMSP-metabolizing microorganisms, such as that previously described for *Octadecabacter* [58]. Additionally, another probable competitor for *Sulfitobacter* in Chile Bay (and WAP in general) could be the eukaryote *Phaeocystis antarctica*, which is a strong DMSP producer in low salinity under freezing conditions [75]. *Phaeocystis antarctica* was present in Chile Bay in 2016 (Figure S1), as was previously reported during other summers at this location [24,25], suggesting a ubiquitous presence of this taxa in the summer coastal waters of Chile Bay. Moreover, *Phaeocystis* has been used as a sentinel for salinity changes in ice [76], as it has an efficient the photosystem and photo-damage repair mechanisms enabling it to grow in high-PAR and well-mixed waters [77,78]. In a similar way, our data demonstrate that *Phaeocystis* experience increased gene expression (up to 60%) when facing dilution in the microcosms, denoting good performance of the Calvin cycle and photosynthesis (Figure 3), as well as for ammonium assimilation and nitrite reduction (Figure 4).

Additionally, two seawater taxa that were hardly detected in the 16S rRNA data showed increased gene expression after seven days in the diluted incubations. The first was HTCC2207 (belonging to the SAR92 group, Gammaproteobacteria), which exhibited increased gene expression (3%) in the diluted experiments compared to the SW control. This Gammaproteobacteria is an important global component of the marine sulfur cycle and has been described as the most relatively abundant bacterioplankton group of the northern Antarctic Peninsula [58]. The second taxon was *Pseudoalteromonas*, which showed a similar pattern response to HTCC2207. It is also a unique marine genus that exhibited a marked increase of activity in metabolic pathways associated with carbon metabolism and fixation (Figure 2c and Figure 5). This important bacterium comprises up to 6% of the total free-living seawater community in polar regions [79], being important to biofilm formation among the dominant community members living on ocean particles [80]. Moreover, species of *Pseudoalteromonas* are generally associated with marine eukaryotes and display antibacterial, bacteriolytic, agarolytic and algicidal activities [81]. Gene expression patterns showed that pathways related to fatty acids, antibiotic compounds and biosynthesis of secondary metabolites were most active in this bacterium under the dilution conditions. Probably the vast capacity of *Pseudoalteromonas* to produce extracellular polymeric substances (EPS), as observed in the ice brine of the Arctic [82,83], could confer to this organism the potential to tolerate and even improve its metabolic fitness during freshening events.

Conversely, some dominant members of the glacier ice, such as *Flavobacterium* spp., decreased in abundance and gene expression by the end of the 7-day experiment. Our results show that carbon metabolism experienced a notable decrease in expression, together with ABC transporters, protein machinery and RNA degradation. As *Flavobacterium* is a widespread taxon, including in polar systems, our results indicate that, despite exhibiting lower expression of important metabolic pathways during the experiment, it is able to maintain certain activity while potentially tolerating saltier water. Similarly, *Polaromonas* showed lower activity of the same metabolic pathways; however, this only represented a 2-fold decrease compared to the >10-fold decrease seen for *Flavobacterium* (Figure 5).

In addition to bacterial sentinels, we detected some dominant photosynthetic eukaryotes that could potentially be used as sentinels, namely Bacillariales, Chaetocerotales and Thalassiosirales. These groups are relevant to maintaining primary production and Chl *a* in the WAP [10,57], and Chile Bay seawater is no exception. Our dilution experiment results showed a minimal or null decrease in their activity, especially with respect to carbon pathways (Figure 3). The potential resilience of these eukaryotic microorganisms when faced with salinity dilution events highlight them as highly relevant indicator organisms for present and future primary production changes throughout the WAP. This is even more important if the current climate warming events continue as predicted [2,5,7].

Here, we demonstrate the potential resilience and collapse of some of the most dominant marine bacteria when facing abrupt changes in salinity, supporting their use as potential sentinels for glacier melting in Chile Bay and possibly throughout the WAP.

## 5. Conclusions

We identified a slight salinity gradient from the glacier discharge to the offshore marine stations in Chile Bay waters. The discharge of freshwater from the glaciers interferes with the supply of nutrients and Chl *a* to the seawater. Among the Eukarya and Bacteria domains, Bacteria demonstrated changes in composition and activity both in the natural system and under manipulated dilution experiments. Potential bacterial sentinels from ice were revealed to represent freshening in the bay. For example, in marine coastal areas surrounded by glaciers, *Flavobacterium* could serve as a warning for a high melting face and freshening input to the marine system, whose melted glacier ice intrusion seems to be highly tolerable by dominant marine bacteria that have metabolic resilience to these changes.

Additionally, through these dilution experiments, several relevant and common marine genera (bacteria and eukaryotes) reacted positively by increasing the expression of hundreds of genes in response to salt dilution. During salinity changes in Chile Bay due to glacier freshening, our results strongly suggest bacterial functional resilience (e.g., *Polaribacter*, *Pseudoalteromonas*, HTCC2207) or tolerance accompanied by a rapid decrease in gene expression (e.g., *Flavobacterium*). Moreover, the positive behavior of eukaryotes during salt dilution could help us to model these events. These results also provide information about the maintenance of the primary and secondary productivity of Chile Bay and, possibly, all coastal waters of the WAP, where freshening events occur throughout the summer. This is of great importance for all Antarctic coastal ecosystems since potential sentinels are the main microorganisms involved in carbon and nitrogen biogeochemical cycles.

## Supplementary Materials

The following are available online at https://www.mdpi.com/2076-2607/9/1/88/s1, Table S1. Average nutrient concentrations observed for different seawater stations (P1, P2 and P3) and melted glacier ice samples. ±: standard deviation. Table S2: Nutrients observed at the beginning (t1) and final (t7) time of the dilution experiments. Figure S1. Eukaryotic members derived from 16S rRNA gene sequencing (classified chloroplast reads) of the in situ salinity transect and glacier ice samples. Figure S2. Heatmap of the 1000 most active eukaryotic, bacterial and archaeal genes in each dilution experiment: SW+ICE, SW+ICE-F and the ICE and SW controls. The pie chart indicates the average percentage of each domain in the dilution and control experiments.
